# Does Second Trimester Maternal Serum Zonulin Level Predict Gestational Diabetes Mellitus?

**DOI:** 10.3390/jcm13020394

**Published:** 2024-01-11

**Authors:** Nazan Vanlı Tonyalı, Burak Arslan, Serap Topkara Sucu, Kemal Sarsmaz, Müjde Can İbanoğlu, Gökçen Örgül, Gizem Aktemur, Aykan Yücel, Dilek Şahin

**Affiliations:** 1 Department of Obstetrics and Gynecology, Division of Perinatology, Health Sciences University Ankara Etlik City Hospital, Ankara 06010, Turkey; drgizemkizilbuga@gmail.com; 2 Department of Psychiatry and Neurochemistry, Institute of Neuroscience and Physiology, The Sahlgrenska Academy at the University of Gothenburg, 42130 Mölndal, Sweden; burak.arslan@gu.se; 3 Department of Obstetrics and Gynecology, Health Sciences University Ankara Etlik City Hospital, Ankara 06010, Turkey; serap.ege@saglik.gov.tr; 4 Department of Obstetrics and Gynecology, Manisa Celal Bayar University, Manisa 45120, Turkey; drsarsmazkemal@gmail.com; 5 Department of Obstetrics and Gynecology, Etlik Zübeyde Hanım Gynecology Training and Research Hospital, Ankara 06010, Turkey; drmujdecan@gmail.com; 6 Department of Obstetrics and Gynecology, Selçuk University, Konya 42100, Turkey; gokcenorgul@gmail.com; 7 Department of Obstetrics and Gynecology, Division of Perinatology, Health Sciences University Ankara Bilkent City Hospital, Ankara 06800, Turkey; aykanyucel@gmail.com (A.Y.); dilekuygur@gmail.com (D.Ş.)

**Keywords:** zonulin, gestational diabetes mellitus, gestational diabetes mellitus therapy

## Abstract

Zonulin, a protein that regulates intestinal permeability, has attracted attention as a potential biomarker for GDM. Therefore, this study aims to investigate whether there are differences in zonulin levels between the GDM group and control groups, especially between those receiving different treatments (diet and insulin). Based on this idea, we included 90 patients with a gestational age between 24 and 28 weeks in our study. While GDM was not detected in 33 of these patients, as a result of OGTT, 57 patients were diagnosed with GDM and these patients were followed throughout their pregnancy. Gestational diabetes was diagnosed by an OGTT performed between 24 and 28 weeks of gestation according to American Diabetes Association (ADA) standards. During follow-up, GDM patients were divided into two groups according to whether they required insulin treatment. Plasma zonulin levels were determined using enzyme-linked immunosorbent assay (ELISA) techniques. The GDM group had significantly higher plasma zonulin levels than the control group (*p* < 0.005). According to our research, zonulin may be a non-invasive biomarker involved in the etiology of GDM. Large-scale research on this topic is still needed.

## 1. Introduction

Gestational diabetes mellitus (GDM) is a common medical complication of pregnancy, affecting approximately 7% of all pregnancies worldwide [1]. It is characterized by high blood glucose levels that develop during pregnancy and, if left unchecked, can lead to a number of adverse outcomes for both mother and child, including increased risk of preeclampsia, preterm birth, macrosomia, and neonatal hypoglycemia [2]. Despite extensive research, the exact mechanisms underlying the development of GDM remain poorly understood. Recently, however, there has been an increased interest in the possible role of the gut microbiome and gut permeability in the development of GDM [3].

Zonulin is a protein that regulates intestinal permeability by controlling the opening and closing of tight junctions between intestinal epithelial cells [4]. Elevated zonulin levels have been found in a number of conditions, including inflammatory bowel disease, celiac disease, and type 1 diabetes [5]. Zonulin has recently emerged as a potential biomarker for GDM, as several studies have reported that women with GDM have higher circulating zonulin levels than women with normal glucose tolerance [6]. The role of zonulin in the development of GDM has not been fully elucidated. However, it is believed that elevated zonulin levels lead to increased intestinal permeability, allowing toxins and other inflammatory mediators to enter the bloodstream, which may contribute to insulin resistance and the development of GDM [6]. In addition, zonulin has been shown to directly affect glucose metabolism by influencing the expression of genes involved in glucose homeostasis [5]. Although there are many studies investigating the relationship between zonulin levels and GDM, there are no current studies comparing zonulin levels in patients with GDM under different treatments. Understanding the differences in zonulin levels between patients on a diet and those taking insulin may shed light on the mechanisms of pathogenesis of GDM and potential treatment approaches. 

The exact mechanisms by which insulin therapy might contribute to increased zonulin levels in GDM are not fully understood. One possibility is that insulin therapy leads to changes in the gut microbiota, which in turn could increase gut permeability and zonulin levels [7]. Alternatively, insulin therapy could directly affect intestinal permeability through its effects on glucose metabolism and inflammation. Overall, while further research is needed to fully elucidate the relationship between zonulin levels and different treatment modalities in GDM, these studies suggest that zonulin may be a useful biomarker for monitoring the efficacy of different treatment approaches in GDM. With this information in mind, we aimed to determine in this study whether there was a difference between zonulin levels in the GDM group compared with the control groups and the different treatments (diet versus insulin).

## 2. Materials and Methods

Study Design: This study was conducted prospectively between February 2020 and November 2020 at the Perinatology Clinic of the Turkish Ministry of Health, Zübeyde Hanım Gynecology and Obstetrics Training and Research Hospital. Written informed consent was obtained from all subjects who participated in this study. This study was conducted in accordance with the principles of the Declaration of Helsinki and approved by the ethics committee of the hospital (approval number: 2020/36).

Patient Selection: Patients were diagnosed with GDM according to the recommendations of the American Society of Obstetrics and Gynecology (ACOG) and the International Association of Diabetic Pregnancy Working Groups (IADPSG) [8]. From 24 to 28 weeks of gestation, all participants were screened for GDM with the 50 g OGTT. In patients whose 1 h glucose level on the 50 g OGTT was ≥140 mg/dL, we used the 100 g OGTT after at least 8 h of fasting. Diagnostic criteria were based on the American Diabetes Association guidelines, including the following: fasting blood glucose level of >95 mg/dL, 1 h blood glucose level of ≥185 mg/dL on the 100 g OGTT, 2 h blood glucose level of >155 mg/dL, and 3 h blood glucose level of ≥140 mg/dL [9]. A positive test for the diagnosis of GDM was defined as two or more high glucose levels at or above these thresholds. Pregnant women diagnosed with GDM were offered appropriate treatment.

The inclusion criteria were as follows: pregnant women diagnosed with GDM in the second trimester (between 24 and 28 weeks of gestation), women aged between 18 and 40 years, women with singleton pregnancy, and women who gave their informed consent to participate in this study. The exclusion criteria were as follows: women with a history of diabetes or other metabolic disorders, gastrointestinal disease or inflammatory bowel disease, autoimmune disease, use of antibiotics or probiotics in the past 4 weeks, fetal abnormalities, or intrauterine growth restriction.

After obtaining informed consent, a detailed history and clinical examination were performed on each participant. Demographic data (age, body mass index (BMI), parity, gestational age, and obstetric history), details of GDM diagnosis (glucose tolerance test results and treatment received), dietary information and adherence, insulin therapy details (type, dose, and frequency), and maternal serum zonulin levels (measured in the second trimester at the time of GDM diagnosis) were collected. In addition, 3 mL serum samples from blood samples collected between 24 and 28 weeks from the cases diagnosed with GDM and the control group were filled into Ependorf tubes. During the follow-up of the pregnant women with GDM, they were divided into two groups based on their blood glucose levels: those who were on a diet only (A1GDM) and those who required insulin (A2GDM). A statistical comparison was performed to determine whether there was a difference between the zonulin levels of the three groups (diet-only blood glucose (A1GDM), insulin-regulated blood glucose (A2GDM), and control group).

Analysis of zonulin level: Maternal serum zonulin levels were analyzed using a commercially available enzyme-linked immunosorbent assay (ELISA) (Human Zonulin ELISA Kit, MyBioSource, San Diego, CA, USA). Samples were collected in the morning after an overnight fast. Serum was separated by centrifugation and stored at −80 °C until analysis. All samples were analyzed in duplicate and the mean was used for analysis.

Statistical analysis: Data were analyzed using SPSS software version 25.0 (IBM Corporation, Armonk, NY, USA). Descriptive statistics were used to summarize the demographic and clinical characteristics of the study population. Continuous variables were expressed as mean ± standard deviation (SD) and categorical variables as percentages. The independent *t*-test and one-way analysis of variance (ANOVA) were used to compare mean serum zonulin levels between groups. A *p*-value of less than 0.05 was considered statistically significant.

Sample size calculation: Sample size calculation was performed using G*Power software version 3.1.9.7. With an alpha error of 0.05, a power of 80%, and a ratio of 1:1:1 between the three groups, the minimum sample size required was estimated to be 14 patients in each group. In our study, a larger sample was obtained than originally planned. Instead of the 42 patients originally considered necessary, 33 patients without GDM, 36 patients with diet-regulated GDM (A1GDM), and 21 patients with insulin-regulated GDM (A2GDM), a total of 90 patients, were enrolled in this study.

## 3. Results

The demographic characteristics, clinical findings, and laboratory parameters of the pregnant women are summarized in Table 1. Analysis of the data revealed no statistical differences between groups in BMI (*p* = 0.146), parity (*p* = 0.053), birth weight (*p* = 0.271), APGAR score (*p* = 0.855), and preterm birth (*p* = 0.048). Maternal age, number of pregnancies, number of live infants, and BMI were significantly higher in the A2GDM group than in the other two groups (Table 1). While there was a significant difference between the history of GDM in previous pregnancies, the control group, and the A2GDM group, there was no difference between the control group and the A1GDM group or between the A1GDM and A2GDM groups. For family history of diabetes, there was a difference between the control group and the two GDM groups, but no difference between the A1GDM and A2GDM groups. For polyhydramnios, there was a significant difference between the control group and the A2GDM group, but no difference between the control group and the A1GDM group or between the A1GDM and A2GDM groups. When zonulin levels were considered, a statistically significant difference was found between the control group and the two GDM groups (*p* < 0.001) (Table 2). The patient group with GDM had higher zonulin levels than the control group. 

**Figure 1:**
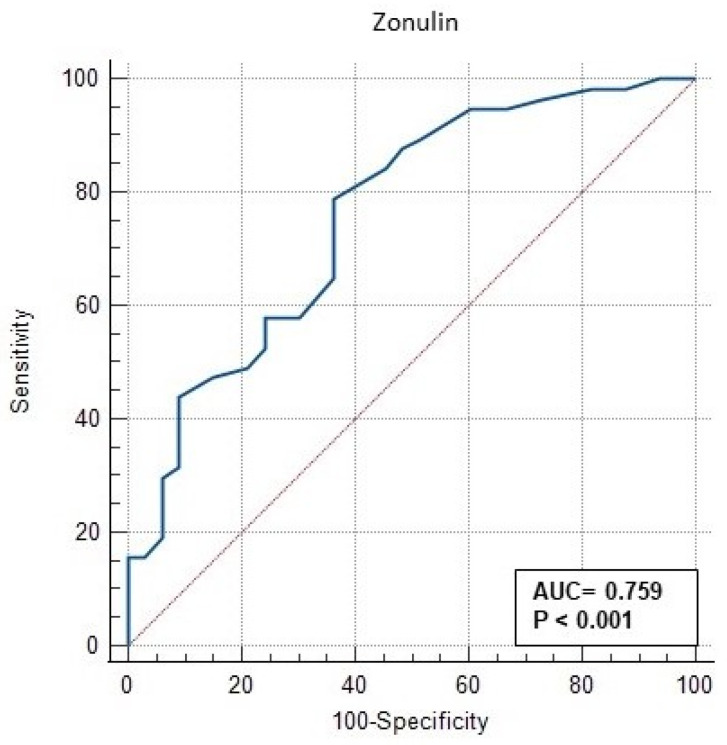
ROC curve of discrimination of zonulin level between both GDM groups and the control group.

**Figure 2:**
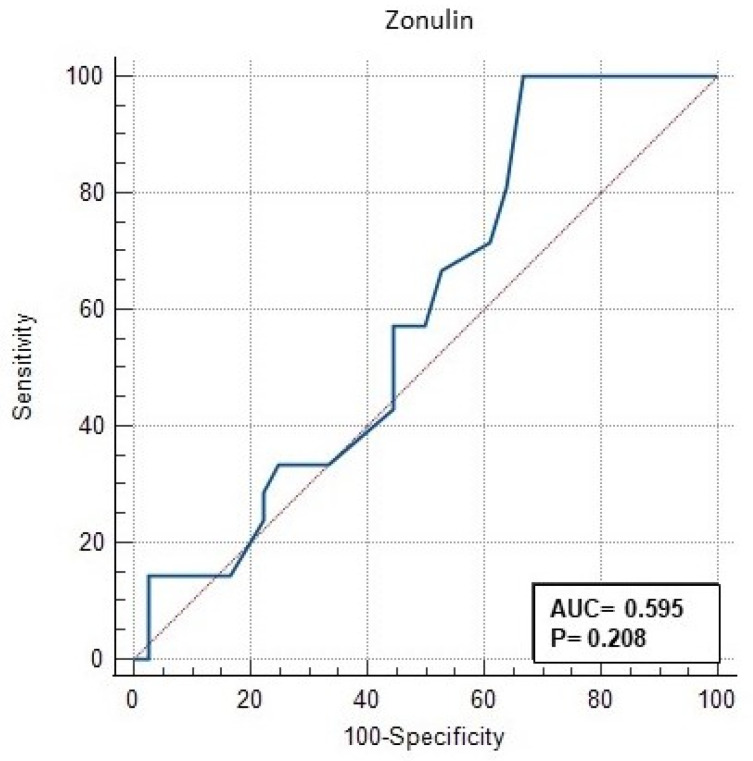
ROC curve of the discrimination of zonulin level between A1GDM and A2GDM.

The ROC (receiver operating characteristic) analysis performed in this study to evaluate the significance of serum zonulin level in GDM is shown in Table 3 and Figure 1. In Figure 1, the AUC (area under the curve) of serum zonulin level was 0.7559 (95% CI: 0.657–0.843) (CI—confidence interval), with a *p* value < 0.001. The ROC curve in Figure 1 shows that the curve for zonulin covers a large area off the baseline, indicating the ability of zonulin to positively predict GDM. Our data suggest that a cut-off value of >20 ng/mL is the value that positively discriminates among women most likely to develop A2GDM (Table 3). This optimal cut-off point was determined by calculating the ROC pairs of sensitivity and specificity and selecting the pair with the smallest distance between them. The sensitivity for these values is 78.95% and the specificity is 63.4%. The positive odds ratio (+LR) (LR—likelihood ratio) was 2.17 and the negative odds ratio (−LR) was 0.33 (Table 3, Figure 1). With a specificity of 90.9% for zonulin, the cut-off value was 28 ng/mL. Using PPV or NPV, this study showed that zonulin level can be used to effectively predict those who are likely to develop A2GDM. However, serum zonulin level was found to have no significant difference in discriminating between A1GDM and A2GDM groups (AUC = 0.595, 95% CI: 0.457–0.723, *p* = 0.208) (Table 4, Figure 2).

## 4. Discussion

Gestational diabetes mellitus (GDM) is a glucose tolerance disorder that occurs during pregnancy and has adverse effects on maternal and fetal health [9]. In our study, we investigated the association between zonulin levels between 24 and 28 weeks of gestation and the development of GDM later in pregnancy. Our study showed that women who later developed GDM had significantly higher serum zonulin concentrations between 24 and 28 weeks of gestation than women who remained normoglycemic throughout the gestational period. The aim of this study was to evaluate the significance of serum zonulin concentrations in distinguishing GDM and in differentiating between diet-controlled and insulin-controlled groups in patients with GDM. This finding is consistent with studies showing increased zonulin levels in people with GDM and type 2 diabetes mellitus [10]. The possible mechanism by which zonulin may play a role in the development of GDM is that it impairs insulin receptor function and stimulates inflammation. In addition, when the intestinal barrier is breached, infectious pathogens and antigens from food can enter the body via mucosal immune elements, leading to increased immune responses and the destruction of pancreatic beta cells and possibly increased cytokine production [10]. Therefore, an increase in zonulin levels is expected to lead to an increase in proinflammatory adipocytokines such as leptin [11]. A previous study has shown that leptin levels increase in the first trimester in women who later develop GDM.

Zonulin is a protein molecule involved in the regulation of intercellular tight junctions and generally plays an important role in maintaining the integrity of the intestinal barrier [2]. This protein is necessary to increase or decrease the permeability of this barrier and thus regulates the entry of macromolecules, microorganisms, and toxins from the intestine into the general circulation [2]. In recent years, evidence has emerged that overproduction of zonulin plays a role in the development of a number of diseases. In particular, high levels of zonulin have been associated with celiac disease, type 1 and type 2 diabetes, obesity, polycystic ovary syndrome, and a number of other inflammatory and autoimmune diseases [12]. These diseases are often characterized by increased intestinal permeability, making zonulin a potential biomarker for better understanding the pathogenesis of these diseases [13]. Our results showed that serum zonulin level was a discriminator between patients with and without GDM (AUC = 0.7559, 95% CI: 0.657–0.843, *p* < 0.001). Notably, patients with a serum zonulin level of >20 ng/mL were found to be more likely to have GDM. These results suggest that serum zonulin level has some discriminatory power in distinguishing GDM and that a value of >20 ng/mL may be a useful cut-off value for accurately predicting the disease. These findings are also supported by data from other studies. For example, Zhang et al. found that serum zonulin level was an independent risk factor for determining the risk of GDM [14]. In addition, Moreno-Navarrete et al. (2012) showed that zonulin is associated with insulin resistance and diabetes [15].

Zonulin level seems to be used as a screening test in the diagnosis of gestational diabetes mellitus (GDM). The data from our study suggest that zonulin level has a good AUC (area under the curve) (0.7559) in detecting GDM, indicating that zonulin level is a good combination of sensitivity and specificity. At a cut-off value of 20 ng/mL, the sensitivity of the test was 78.9% and the specificity was 63.6%. This indicates that zonulin level has a high ability to accurately detect the presence of GDM, but there is still a possibility of false-positive results. In contrast, the specificity of the test increased to 90.9% at a cut-off value of 28 ng/mL, but the sensitivity decreased. This shows that with its cut-off value, the test can be a very effective screening test to accurately detect those without GDM and that at this value, a diagnostic glucose load test can be performed in pregnant women with higher zonulin levels. GDM is an important public health problem worldwide and in Turkey [16]. According to the World Health Organization (WHO), the global prevalence of GDM varies between 2 and 10%, while the rate in Turkey is 7.7% (range: 1.9–27.9%) according to 2019 data [16]. In view of these data, our country belongs to the group of countries with a higher prevalence of GDM, and therefore screening for GDM is becoming increasingly important [16]. Compared with the 50 g oral glucose tolerance test (OGTT), zonulin level measurement is a less invasive test [6]. In recent years, discussions in the media about the OGTT in Turkey have significantly influenced the preference of pregnant women for this test [17]. Following these discussions, many pregnant women avoid this test due to concerns about the potential risks of the test and their perception that the OGTT may harm them or their baby [18]. This has led to a significant decrease in the number of pregnant women who accept the OGTT. The results of the phenomenon, which Hoffman et al. called the “celebrity effect”, are also supported by studies [19]. According to studies, especially after 2014, there was a serious prejudice against the 50 g OGTT in Turkish society due to some unfounded statements in the media that the 50 g OGTT had serious side effects on the fetus, so the number of pregnant patients undergoing these screening tests has decreased in recent years [20]. A dramatic decline has been observed. In a study conducted in 2018 in a third-level center in our country, while the rate of pregnant women who agreed to have a sugar challenge test was 7.55% in 2013, this rate decreased to 3.9% in 2017 [21]. In addition, there are pregnant women who do not want to have these tests because they had mild side effects (nausea, vomiting, diarrhea) in their previous pregnancies after a 50 g OGTT [22]. For all these reasons, it is important to evaluate alternative screening tests in societies with a high prevalence of GDM, as in our country. However, the OGTT is the most sensitive test accepted and is used for GDM screening [18]. Further research is needed for the screening and diagnostic use of serum zonulin levels, particularly to reduce the false-negative rate, determine the optimal cut-off value, and improve the overall performance of the test. In addition, how zonulin levels interact with other demographic and clinical variables and how to manage these interactions should be investigated.

However, in this study, the power of serum zonulin levels to discriminate treatment strategies in GDM patients was limited (AUC = 0.595, 95% CI: 0.457–0.723, *p* = 0.208). Zonulin levels did not differ in the treatment approach of patients with GDM, i.e., no significant difference in zonulin levels was observed between GDM patients who could be controlled by diet and those who required insulin therapy. In contrast to some studies showing that the association between zonulin levels and GDM is consistent, several studies have not confirmed this association [23]. These inconsistencies are thought to be due to studies conducted in different populations and in individuals of different ages. This suggests that further studies are needed to fully understand the effects of zonulin levels on the pathophysiology of GDM. The use of zonulin for screening and diagnosis of gestational diabetes mellitus (GDM) has potential limitations. Some of these include the problem of consistency and comparability between laboratories because there is no standard method for measuring zonulin levels, the test is expensive and less accessible compared to more common and less expensive traditional GDM screening and diagnostic tests, and there is a lack of validation studies. These factors may limit the use of zonulin as a screening and diagnostic test for GDM. However, as more research is conducted on this topic, some of these limitations may be overcome.

## 5. Conclusions

The results of our study suggest that zonulin may be a potential biomarker for the diagnosis of GDM and that it has limited utility in discriminating between different treatment options. However, further investigation and studies with larger samples may help us to better understand the role of zonulin in the pathogenesis of GDM and the efficacy of its clinical application. In addition, ROC analysis of the relationship between zonulin levels and GDM in this study showed that the ability of zonulin levels to discriminate GDM was average. However, these results should be interpreted with caution. ROC analysis can evaluate the ability of a biomarker to detect disease, but this does not necessarily mean that it is effective in clinical practice [6]. Therefore, further studies are needed to evaluate the effectiveness of zonulin level in clinical practice.

However, this study has some limitations. First, our study population included only a limited number of patients. Therefore, larger studies are needed to provide further insight into the ability of serum zonulin level to determine GDM and treatment strategies in a larger sample. Second, only serum zonulin levels were examined in this study, so the role of other potential biomarkers in the pathophysiology and treatment of GDM was not a focus.

In conclusion, the results of this study suggest that zonulin level may be a potential biomarker for the diagnosis of GDM. However, further studies are needed to evaluate its usefulness in clinical application.

## Figures and Tables

**Table 1:** Pregnancy characteristics of the study population.

	A1GDM (N = 36)	A2GDM (N = 21)	Control (N = 33)	Totals (N = 90)	*p*
Age, years mean ± SD	30.8 ± 5.71a	34.1 ± 4.49b	30.4 ± 5.70a	31.4 ± 5.58	0.047 *
Body Mass Index (kg/m^2^), mean ± SD	30.3 ± 5.36	32.2 ± 4.18	29.8 ± 3.61	30.6 ± 4.56	0.146 *
Parity, N, median (IQR)	2 (1–4)a	3 (3–4.5)b	2 (2–4)a	3 (2–4)	0.048 **
Gestational Age, days mean ± SD	180 ± 7.9	182 ± 5.5	180 ± 7.3	181 ± 7.1	0.801 *
History of GDM, n (%)	5 (13.9)ab	6 (28.6)a	0 (0)b	11 (12.2)	0.007 ***
Family DM History, n (%)	17 (47.2)a	13 (61.9)a	8 (18.2)b	36 (40)	0.003 ***

Abbreviations: A1GDM—diet-controlled gestational diabetes; A2GDM—insulin controlled gestational diabetes; IQR—interquartile range; DM—diabetes mellitus; GA—gestational age; SD—standard deviation; n—number; kg—kilogram; m^2^—square meter. Mean values marked with different letters differ significantly. Data expressed as n (%), median (minimum–maximum), or mean (± standard deviation). * ANOVA test (Post-hoc test: Bonferroni correction), ** Kruskal–Wallis (Bonferroni correction Mann–Whitney U test), *** Chi-square test.

**Table 2:** Laboratory values and pregnancy outcomes of the study groups.

	A1GDM (N = 36)	A2GDM (N = 21)	Control (N = 33)	Totals (N = 90)	*p*
50 g GGT 1. Hour, mg/dL median (IQR)	181.5b (160–193.25)	167b (161–194)	103a (85.5–121.5)	162 (117.25–188.25)	<0.001 *
Hb a1c, g/dL median (IQR)	5.5 (5.1–5.8)	5.8 (5.6–6.2)	-	5.67 (5.4–6.07)	0.008 *
Mode of birth n (%) VD	27 (75)	16 (76.2)	22 (66.7)	65 (72.2)	0.667 **
CS	9 (25)	5 (23.8)	11 (33.3)	25 (27.8)	
Neonatal birth weight grams, median (IQR)	3270 (2935–3465)	3080 (2957.5–3370)	3265 (3077.5–3520)	3250 (2977.5–3465)	0.271 *
Apgar 1 min, median (IQR)	9 (9–9)	9 (9–9)	9 (9–9)	9 (9–9)	0.855 *
Apgar 5 min, median (IQR)	10 (10–10)	10 (10–10)	10 (10–10)	10 (10–10)	0.855 *
Zonulin, ng/mL median (IQR)	25.5a (19.25–33.75)	28a (22–35.5)	18b (14–25)	23 (17.75–29.25)	<0.001 *
Preterm delivery (<37 weeks) n (%)	3 (8.3)	1 (4.8)	2 (6.1)	6 (6.7)	0.89 **
Polyhydramnios, n (%)	2 (5.6)ab	5 (23.8)a	1 (3)b	8 (8.9)	0.045 **

Abbreviations: A1GDM diet—controlled gestational diabetes; A2GDM—insulin controlled gestational diabetes, HbA1C—hemoglobin A1C; C/S—cesarean section; GTT—glucose tolerance test; SD—standard deviation; n—number; mg—milligram; dL—deciliter; L—liter; g—gram; ng—nanogram; IQR—interquartile range.; VD—vaginal delivery. Mean values marked with different letters differ significantly. Data expressed as n (%), median (minimum–maximum), or mean (± standard deviation). * Kruskal–Wallis (Bonferroni correction Mann–Whitney U test), ** Chi-square test.

**Table 3:** Receiver operating characteristic (ROC) curve analysis for the discrimination of zonulin level between both GDM groups and the control group.

	AUC	95% CI	*p*	Cut-Off	Sensitivity (%)	Specificity (%)	+LR	−LR
Zonulin Level	0.7559	0.657–0.843	<0.001	>20	78.95	63.64	2.17	0.33

AUC—area under the curve; LR—likelihood ratio; CI—confidence interval.

**Table 4:** Receiver operating characteristic (ROC) curve analysis for the discrimination of zonulin level between A1GMD and A2GDM.

	AUC	95% CI	*p*	Cut-Off	Sensitivity (%)	Specificity (%)	+LR	−LR
Zonulin Level	0.595	0.457–0.723	0.208	>20	100	33.33	1.5	0

AUC—area under the curve; LR—likelihood ratio; CI—confidence interval.

## Data Availability

On reasonable request, the corresponding author will provide the information supporting this study’s conclusions.

## References

[1] Etminan-Bakhsh M., Tadi S., Hatami M., Darabi R. (2020). Prevalence of Gestational Diabetes Mellitus and Its Associated Risk Factors in Boo-Ali Hospital, Tehran. Galen Med. J..

[2] Plows J.F., Stanley J.L., Baker P.N., Reynolds C.M., Vickers M.H. (2018). The pathophysiology of gestational diabetes mellitus. Int. J. Mol. Sci..

[3] Fasano A. (2020). All disease begins in the (leaky) gut: Role of zonulin-mediated gut permeability in the pathogenesis of some chronic inflammatory diseases. F1000Research.

[4] Tripathi A., Lammers K.M., Goldblum S., Shea-Donohue T., Netzel-Arnett S., Buzza M.S., Antalis T.M., Vogel S.N., Zhao A., Yang S. (2009). Identification of human zonulin, a physiological modulator of tight junctions, as prehaptoglobin-2. Proc. Natl. Acad. Sci. USA.

[5] Wood Heickman L.K., DeBoer M.D., Fasano A. (2020). Zonulin as a potential putative biomarker of risk for shared type 1 diabetes and celiac disease autoimmunity. Diabetes/Metab. Res. Rev..

[6] Demir E., Ozkan H., Seckin K.D., Sahtiyancı B., Demir B., Tabak O., Kumbasar A., Uzun H. (2019). Plasma zonulin levels as a non-invasive biomarker of intestinal permeability in women with gestational diabetes mellitus. Biomolecules.

[7] Mokkala K., Röytiö H., Munukka E., Pietilä S., Ekblad U., Rönnemaa T., Eerola E., Laiho A. (2016). Gut Microbiota Richness and Composition and Dietary Intake of Overweight Pregnant Women Are Related to Serum Zonulin Concentration, a Marker for Intestinal Permeability. J. Nutr..

[8] Hod M., Kapur A., McIntyre H.D., Committee PoeN (2019). Evidence in support of the International Association of Diabetes in Pregnancy study groups’ criteria for diagnosing gestational diabetes mellitus worldwide in 2019. Am. J. Obstet. Gynecol..

[9] Bulletins-Obstetrics C. (2013). Practice Bulletin No. 137: Gestational diabetes mellitus. Obstet. Gynecol..

[10] Zhang D., Zhang L., Zheng Y., Yue F., Russell R.D., Zeng Y. (2014). Circulating zonulin levels in newly diagnosed Chinese type 2 diabetes patients. Diabetes Res. Clin. Pract..

[11] Bawah A.T., Seini M.M., Abaka-Yawason A., Alidu H., Nanga S. (2019). Leptin, resistin and visfatin as useful predictors of gestational diabetes mellitus. Lipids Health Dis..

[12] Vojdani A., Vojdani E., Kharrazian D. (2017). Fluctuation of zonulin levels in blood vs stability of antibodies. World J. Gastroenterol..

[13] Fasano A. (2011). Zonulin and its regulation of intestinal barrier function: The biological door to inflammation, autoimmunity, and cancer. Physiol. Rev..

[14] Zhang H., Wang Q., He S., Wu K., Ren M., Dong H., Di J., Yu Z., Huang C. (2020). Ambient air pollution and gestational diabetes mellitus: A review of evidence from biological mechanisms to population epidemiology. Sci. Total Environ..

[15] Moreno-Navarrete J.M., Sabater M., Ortega F., Ricart W., Fernandez-Real J.M. (2012). Circulating zonulin, a marker of intestinal permeability, is increased in association with obesity-associated insulin resistance. PLoS ONE.

[16] Karaçam Z., Çelİk D. (2021). The prevalence and risk factors of gestational diabetes mellitus in Turkey: A systematic review and meta-analysis. J. Matern.-Fetal Neonatal Med..

[17] Sezer H., Yazici D., Canbaz H.B., Gonenli M.G., Yerlikaya A., Ata B., Bekdemir B., Nalbantoglu E.A. (2022). The frequency of acceptance of oral glucose tolerance test in Turkish pregnant women: A single tertiary center results. North. Clin. Istanb..

[18] Çakir A., Çalik K.Y. (2020). Gebelerin Oral Glikoz Tolerans Testi (OGTT) Yaptırma Durumlarına Medyanın Etkisi. Sürekli Tıp Eğitimi Derg..

[19] Hoffman S.J., Mansoor Y., Natt N., Sritharan L., Belluz J., Caulfield T., Freedhoff Y., Lavis J.N., Sharma A.M. (2017). Celebrities’ impact on health-related knowledge, attitudes, behaviors, and status outcomes: Protocol for a systematic review, meta-analysis, and meta-regression analysis. Syst. Rev..

[20] Özceylan G., Toprak D. (2020). Effects of controversial statements on social media regarding the oral glucose tolerance testing on pregnant women in Turkey. AIMS Public Health.

[21] Karasu Y. (2018). Şeker yükleme testine ne oldu? medyanın halk sağlığı üzerine etkisi. Ank. Eğitim Ve Araştırma Hastan. Tıp Derg..

[22] Uyanıkoğlu H., İncebıyık A., Karakaş Yiğit E. (2016). Is the 50-gram Oral Glucose Tolarence Test (OGTT) Essential for the Screening of Gestational Diabetes. Anadolu Klin Ocak.

[23] Daneshvar M., Yadegari A., Ribaldone D.G., Hasanzadeh M., Djafarian K. (2022). Zonulin levels in complicated pregnancy: A systematic review and meta-analysis. J. Obstet. Gynaecol..

